# Calcitonin negative Medullary Thyroid Carcinoma: a challenging diagnosis or a medical dilemma?

**DOI:** 10.1186/s12902-019-0367-2

**Published:** 2019-05-29

**Authors:** Claudio Gambardella, Chiara Offi, Renato Patrone, Guglielmo Clarizia, Claudio Mauriello, Ernesto Tartaglia, Francesco Di Capua, Sergio Di Martino, Roberto Maria Romano, Lorenzo Fiore, Alessandra Conzo, Giovanni Conzo, Giovanni Docimo

**Affiliations:** Division of General and Oncologic Surgery, Department of Cardiothoracic Sciences, University of Campania “Luigi Vanvitelli”, School of Medicine, Via Sergio Pansini 5, 80131 Naples, Italy

**Keywords:** Medullary thyroid carcinoma, Calcitonin negative, Calcitonin, Carcinoembryonic antigen, Chromogranin a, Procalcitonin, Thyroid nodule

## Abstract

**Background:**

Medullary thyroid carcinoma is a neuroendocrine tumor belonging form a malignant growth of the thyroid parafollicular C-cells, representing from 1 to 10% of all thyroid cancer. The biochemical activity of medullary thyroid carcinoma includes the production of calcitonin and carcinoembryogenic antigen, which are sensitive tumor markers, facilitating the diagnosis, follow-up and prognostication. The diagnosis is reached through the identification of high basal calcitonin serum level or after pentagastrin stimulation test. Medullary thyroid carcinoma is able to produce other relevant biomarkers as procalcitonin, carcinoembryionic antigen and chromogranin A. In Literature are described few cases of medullary thyroid carcinoma without elevation of serum calcitonin, an extremely rare event. The aim of this study was to analyse the presentation, the main features and therapeutic management of medullary thyroid carcinoma associated with negative serum calcitonin levels.

**Methods:**

Using the PubMed database, a systematic review of the current Literature was carried out, up to February 2018. Finally, nineteen articles met our inclusion criteria and were selected according to the modified Newcastle-Ottawa scale.

**Results:**

Fourty-nine patients with definitive pathology confirming medullary thyroid carcinoma and with calcitonin serum level in the normal range were identified (24 female, 24 male and not reported gender in 1 case). Mean age was 51.7 years. Serum calcitonin levels were reported for 20 patients with a mean value of 8.66 pg/mL and a range of 0.8–38 pg/mL. Despite the low or undetectable calcitonin serum level, at immunochemistry in almost the half of the cases reported by the Authors, the tumors presented diffuse or focal positivity for calcitonin and carcinoembryionic antigen, while was reported a chromogranin A positivity in 41 of the 43 tested patients.

**Conclusions:**

Calcitonin negative medullary thyroid carcinoma is an extremely rare pathology. The diagnosis and the surveillance is often challenging and delayed, due to the lack of elevation of serum markers as calcitonin and carcinoembryionic antigen. Further studies are needed, to better define options for management of non secretory medullary thyroid carcinoma and to identify new and reliable biomarkers associated to diagnosis and relapse of this medical dilemma.

## Background

Medullary thyroid carcinoma (MTC) is a neuroendocrine tumor (NET) originating from a malignant growth of the thyroid parafollicular C-cells.

At first described by Hazard et al. in 1959, [1] parafollicular C-cells have a neural crest ectoderm and an ultimobranchial body derivation and account for about 1% of all thyroid cells. They have a neuroendocrine role of paramount importance on the calcium homeostasis throughout the production and the secretion of calcitonin (CT) hormone, a 32-aminoacid linear polypeptide.

MTC represent from 1 to 10% of all thyroid cancer with a mean survival of 8.6 years and a 10-years survival rates ranging from 69 to 89%. It is frequently sporadic (75% of cases), otherwise, in case of RET proto-oncogene germline mutation, it has a hereditary pattern (25% of cases). This familial form belongs to multiple endocrine neoplasia type 2 (MEN 2), which present two subtypes MEN 2A – MTC in combination with pheochromocytoma and hyperparathyroidism – and MEN 2B – MTC with an infancy onset, in association to pheochromocytoma, multiple mucosal neuromas, gastrointestinal ganglioneuromatosis and megacolon.

Sporadic MTC has a low growth rate, is well differentiated and generally present a locally aggressiveness. Familial MTC forms, especially in MEN 2B, present a worse prognosis with earlier lymph nodes metastasis and adjacent structures invasion. Central compartment lymph nodes (IV-VI levels) are frequently involved, followed by levels II to V. [2, 3] Metastatic spread to the upper and anterior mediastinum has been described. Haematogenous dissemination involves liver, lungs and bones, even if distant metastases generally occur as a fine miliary pattern, hardly visualized by computed tomography (CT). [2]

Histologically, typical medullary tumor is characterized by round cells producing amyloid substances, separated by fibrous septa and has microcalcification areas. [1]

The biochemical activity of MTC includes the production of CT and carcinoembryogenic antigen (CEA), which are sensitive tumor markers, related to mass size, facilitating the diagnosis, follow-up and prognostication of MTC. In MTC, CT value is high at basal and after pentagastrin stimulation test, resulting in MTC a high sensitivity and specificity indicator of disease. MTC, as other NET, is able to produce many relevant biomarkers as procalcitonin (proCT) the precursor of calcitonin, neuron specific enolase (NSE) and chromogranin A (CgA). [3] Falsely high or low level of CT are associated with several disease such as C-cells hyperplasia, autoimmune thyroiditis, end stage renal disease, lung and prostate cancer and some neuroendocrine tumors. Otherwise, in patients with millimetric MTC, it is possible to identify normal basal level of CT. It is extremely rare to diagnose voluminous and palpable MTC associated with normal CT level, since, in most cases there is a correlation between size and basal CT level. [4] In 1989, Sobol et al. reported the first case of CT negative MTC, and to date only few cases have been occasionally described in Literature. [5]

The aim of his study was to analyse the presentation, the main features and therapeutic management of MTC patients associated with negative serum CT level.

## Methods

Using the PubMed database, a systematic review of the current Literature was carried out, up to February 2018. The MeSH (Medical Subject Headings) search terms used were “thyroid”, “medullary”, “carcinoma”, “endocrine” and “neuroendocrine tumors”. The Authors observed that MTC CT-negative was an extremely rare neoplasm. The keywords “calcitonin”, “serum calcitonin”, “calcitonin negative”, “thyroid”, “thyroid gland”, “neuroendocrine”, “neuroendocrine tumor”, “medullary thyroid carcinoma” were used for the research. Several combinations of the keywords and MeSH terms were utilized as showed: “Medullary thyroid carcinoma calcitonin-negative”, “MTC without serum calcitonin”, “Neuroendocrine thyroid tumor lack calcitonin”. The various terms were substituted during the search. References of the more relevant articles were manually searched. The last research was concluded on February 1, 2018.

The search was carried out by two Authors CO, CG and the obtained results were discussed with the senior Author GD. The final article was realised in accordance with the Preferred Reporting Items for Systematic Reviews and Meta-Analyses (PRISMA) guidelines. [1] Moreover, the eligible articles were selected according to the modified Newcastle-Ottawa scale in order to satisfy the requirements of the current review. The scale range is from 0 to 9. The studies included were those presenting a score of 6 or higher. [6–8]

The following data were extracted from the included studies: first author, year of data collection, year of publication, country of origin, characteristics of study population, number of patients with MTC CT-negative, clinicopathological characteristics, matching criteria, disease-free survival (DFS) and overall survival (OS).

The inclusion criteria of the study comprised the report of patients with a proven histopathological diagnosis of MTC associated with normal preoperative serum calcitonin, the presence of the evaluation of clinicopathological features and of the analysis of survival. All studies that failed to fulfil the established inclusion criteria and the not English language studies were excluded.

In all the studies, MTC diagnosis was based on the definitive pathology. Microscopically, MTCs features consist of polygonal or fusiform cells, grouped into nests, trabeculae or follicles; in adjacent struma are present amyloid deposits deriving from altered polypeptides of calcitonin. A peculiar characteristic of MTC at an electronic microscope examination, is the presence of electron-bound granules adjacent to the membrane. Histologically, familial MTC can be distinguished from sporadic MTC by the presence of multicentric C-cell hyperplasia in the thyroid parenchyma. The tumor-node-metastasis (TNM) staging system from AJCC was considered for comparison. The clinical characteristics included age, sex, localization of the neoplasm, size, functional hormonal status and presence of symptoms. The OS and DFS of the patients were also analysed.

## Results

**Fig. 1:**
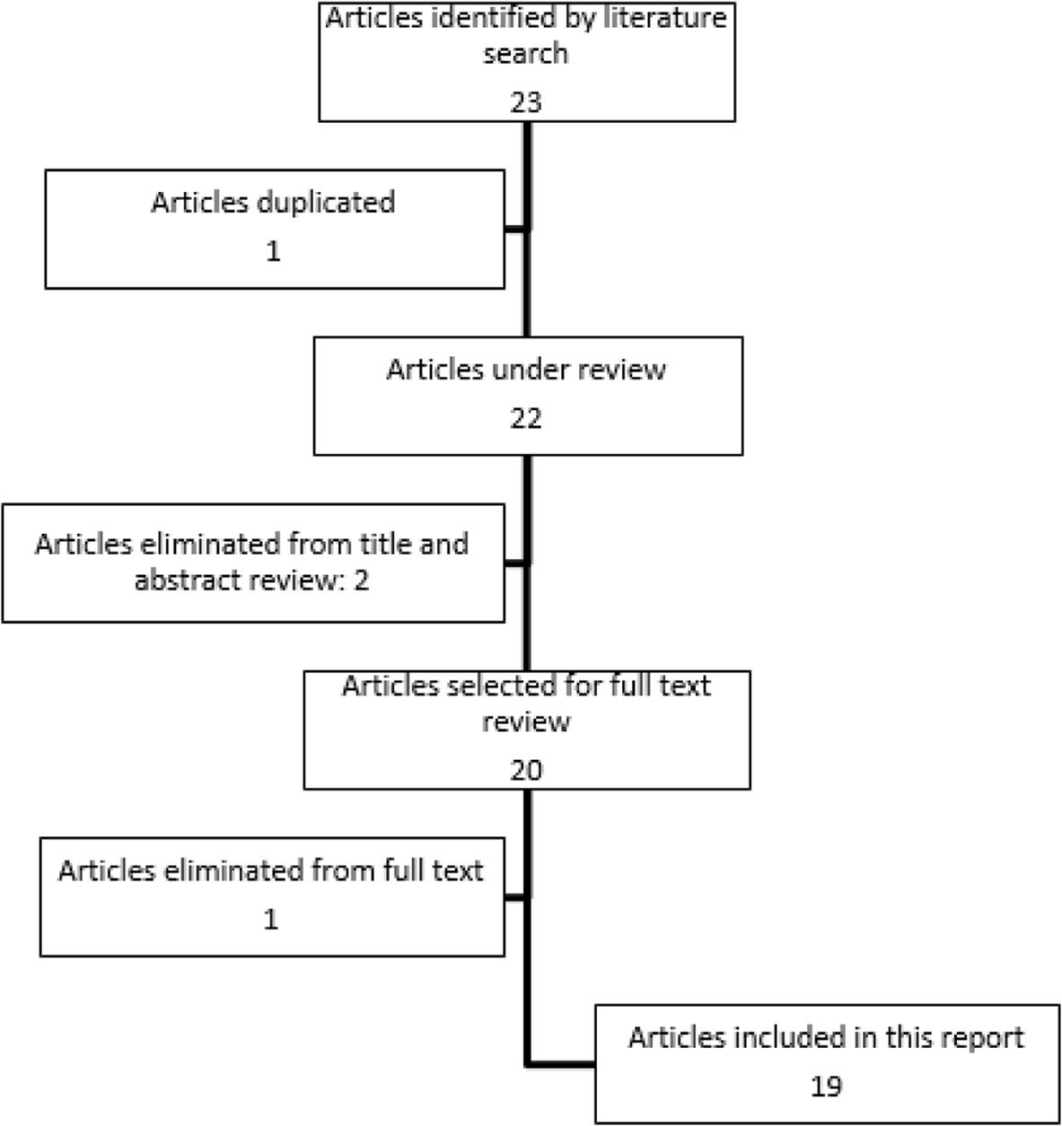
Flow-chart of the papers selection process for CT-negative MTC

Twenty-three suitable studies were identified after Literature review. After the removal of a duplicate study, twenty-two articles were selected for the full-text review. A study was excluded because it was in Spanish (Iglesias P et al. Anaplastic variant of thyroid medullar carcinoma. Med Clin (Barc) 1997). An article was excluded because the MTC diagnosis was made post-mortem (Eusebi V et al. Calcitonin free oat-cell carcinoma of the thyroid gland. Virchows Arch A Pathol Anat Histopathol. 1990). Another one was ruled out because was not possible to recover (Diez JJ. et al. Lack of elevated serum carcinoembryonic antigen and calcitonin in medullary thyroid carcinoma. Thyroid. 2004) and the last was excluded because it did not meet our inclusion criteria (Mussazhanova Z et al. Radiation-associated small cell neuroendocrine carcinoma of the thyroid: a case report with molecular analyses. Thyroid. 2014). [Fig. 1] Therefore, nineteen responded to our inclusion criteria and were enrolled in the current review. The features of the nineteen selected studies were summarized in Table 1.

**Table 1:** Prospectus of the included studies

Author	Reference	Journal	Year
Sobol RE	Hormone-negative, chromogranin A-positive endocrine tumors.	New England Journal of Medicine	1989
Schmid KW	“Atypical” medullary thyroid carcinoma with little or no calcitonin expression.	Virchows Archive A Pathological Anatomy and Histopathology	1998
Redding AH	Normal preoperative calcitonin levels do not always exclude medullary thyroid carcinoma in patients with large palpable thyroid masses.	Thyroid	2000
Bockhorn M	Lack of elevated serum carcinoembryonic antigen and calcitonin in medullary thyroid carcinoma.	Thyroid	2004
Sand M	Serum calcitonin negative medullary thyroid carcinoma.	World journal of surgical oncology.	2006
Dora JM	Normal perioperative serum calcitonin levels in patients with advanced medullary thyroid carcinoma: case report and review of the literature.	Thyroid	2008
Wang TS	Medullary thyroid carcinoma without marked elevation of calcitonin: a diagnostic and surveillance dilemma.	Thyroid	2008
Giovanella L	Serum calcitonin-negative medullary thyroid carcinoma: role of CgA and CEA as complementary markers.	The International Journal of Biological Markers	2008
Alapat DV	Disparity between tissue and serum calcitonin and CEA in patient with medullary thyroid carcinoma.	Endocrine	2011
Chernyavsky VS	Calcitonin-negative neuroendocrine tumor of the thyroid: a distinct clinical entity.	Thyroid	2011
Nakazawa T	C-cell-derived calcitonin-free neuroendocrine carcinoma of the thyroid: the diagnostic importance of CGRP immunoreactivity.	International journal of surgical pathology.	2011
Frank-Raue K	Prevalence and clinical spectrum of nonsecretory medullary thyroid carcinoma in a series of 839 patients with sporadic medullary thyroid carcinoma.	Thyroid	2013
Ismi O	Calcitonin-negative neuroendocrine tumor of thyroid gland mimicking anaplastic carcinoma: an unusual entity.	Gland Surgery	2014
Brutsaert EF	Medullary thyroid cancer with undetectable serum calcitonin.	The Journal of clinical endocrinology and metabolism.	2014
Kim JY	A calcitonin-negative neuroendocrine tumor derived from follicular lesions of the thyroid.	Endocrinology and metabolism.	2015
Kasajima A	A Calcitonin Non-producing Neuroendocrine Tumor of the Thyroid Gland.	Endocrine pathology.	2016
Samà MT	Clinical challenges with calcitonin-negative medullary thyroid carcinoma.	Journal of cancer research and clinical oncology	2016
Parmer M	Calcitonin-Negative Neuroendocrine Tumor of the Thyroid.	International journal of surgical pathology.	2017
Zhou Q	Clinical and pathological analysis of 19 cases of medullary thyroid carcinoma without an increase in calcitonin.	Experimental and toxicologic pathology: official journal of the Gesellschaft für Toxikologische Pathologie.	2017

### Demographic and clinicopathological features

From the selected studies, fourty-nine patients with definitive pathology confirming MTC with a CT serum level in the normal range were identified (24 female, 24 male and unknown gender in 1 case). Mean age was 51.7 years, with a median of 53 years and a range of 16–82 years. Mean cancer size was 63 mm (range 10-80 mm), even if in 7 cases the cancer dimension was not reported. Zhou et al. reported 18 cases of monofocal MTC and one patient showed a multifocal tumor, moreover, 14 patients presented a mass larger than 10 mm of volume and 5 smaller than 10 mm of volume. [9] [Table 2] Eight patients (16.6%) presented laterocervical and central compartment lymph nodes metastases on definitive pathology, [4, 9, 10] while 23 patients (47.9%) did not present lymph nodes metastases and 6 (12.5%) had Nx stage at TNM staging. Other reported sites of MTC metastasis were lung, founded in 4 cases (8.3%), [10, 11] brain, in one case (2%) and lymph nodes of other body districts, found in one case (2%). [11, 12]

**Table 2:** Demographic and clinicopathological features of CT negative MTC patients

	Author	Gender	Age (years)	Size (mm)	Histology	CT-HIC	CT	Assay	Upper reference limit
1	Sobol	F	82	20	WDMTC	Negative	Normal limits	NA	NA
2	Schmid 1	M	28	NA	WDMTC	Weak	NA	Immunotech	NA
3	Schmid 2	M	46	NA	WDMTC	Weak	NA	Immunotech	NA
4	Schmid 3	F	45	NA	WDMTC	Weak	NA	Immunotech	NA
5	Schmid 4	M	37	NA	WDMTC	Negative	NA	Immunotech	NA
6	Redding	F	31	45	WDMTC	Diffuse	28	RIA	< 150
7	Bockhorn	F	50	20	PDMTC	Weak	0.8	Nichols	< 4.6
8	Sand	F	73	NA	PDMTC	Weak	5.3	Nichols	< 10
9	Dora	M	43	20	PDMTC	Diffuse	4	Immunolite	< 12
10	Wang	M	68	70	WDMTC	Weak	38	NA	< 10
11	Giovanella	F	43	48	WDMTC	Diffuse	4.7	Immunolite	< 10
12	Alapat	F	16	30	WDMTC	Diffuse	4	Immunolite	< 4.6
13	Chernyavsky	F	40	20	WDMTC	Negative	2.1	Ventana Medical System Inc.	< 5.0
14	Nakazawa	M	76	60	WDMTC	Weak	22	NA	< 10
15	Frank-Raue 1	F	61	10	WDMTC	Weak	2.9	Nichols	< 10
16	Frank-Raue 2	M	70	80	WDMTC	Weak	< 2	DiaSorin	< 6.1 male < 3.6 female
17	Frank-Raue 3	F	50	20	WDMTC	Weak	0.8	Nichols	< 10
18	Frank-Raue 4	M	47	30	PDMTC	Focal	2.6	Immunolite	< 8.4 male < 5 female
19	Frank-Raue 5	F	53	45	WDMTC	Diffuse	NA	NA	NA
20	Frank-Raue 6	M	45	18	PDMTC	Weak	11	Non commercial	< 18
21	Frank-Raue 7	F	45	55	PDMTC	Focal	1.5	CIS	< 10
22	Ismi	NA	57	NA	PDMTC	Negative	5.6	NA	< 10
23	Brutsaert	F	49	26	WDMTC	Diffuse	< 2	NA	< 6
24	Kim	M	34	10	PDMTC	Negative	3.7	NA	< 10
25	Kasajima	F	48	30	WDMTC	Negative	29	NA	NA
26	Samà 1	M	60	38	NA	Focal	7.8	NA	NA
27	Samà 2	F	66	NA	NA	NA	5	NA	NA
28	Samà 3	M	53	12	NA	Negative	< 10	NA	NA
29	Samà 4	M	62	45	NA	Focal	13	NA	NA
30	Parmer	F	74	20	WDMTC	Negative	Normal limits	NA	NA
31–49	Zhou	11 M 8 F	≥30 3 cases; < 30 16 cases	≤10mmm 14 cases > 10 mm 5 cases	NA	Positive in 8 cases Negative in 11 cases	NA	NA	NA

(*F* female, *M* male, *WDMTC* well differentiated MTC, *PDMTC* poorly differentiated MTC, *CT* calcitonin, *CT-IHC* immunohistochemistry for CT, *NA* Not Avaible)

The clinical presentation, not reported in all cases, were the presence of a palpable mass (11 cases), neck pain (3 cases), an incidentaloma on US (2 cases), shortness of breath (2 cases), cervical lymphadenopathy (1 case), loss of weight (1 case), dysphonia (1 case), paralysis of the ipsilateral vocal cord (1 case), dysphagia (1 case) and diarrhea (1 case).

Only one case presented familiarity with thyroid cancer, [13] even if in 10 cases data on familiarity were not reported. The pathological anamnesis showed 3 patients (6.25%) suffering of thyroid diseases: a case of Hashimoto’s thyroiditis, [14] a case of iatrogenic hyperthyroidism following amiodarone assumption [15] and one of non-toxic multinodular goiter. [12] Furthermore, we found that two patients (4%) were diagnosed with prostate cancer [15] and with breast cancer [16] respectively.

### Preoperative evaluation and surgery

Regarding preoperative serum hormones levels, CT levels were reported only for 20 patients with a mean value of 8.66 pg/mL and a range of 0.8–38 pg/mL. (Table 3) In two cases normal CT levels were founded, however, the assay utilized and the considered range values were not specified. [5–10] In one case, it was reported a value of preoperative CT ≤2 pg/mL, [10] in a case a value of < 0.8 pg/mL, [10] in a case a value of < 10 pg/mL [4] and in 5 cases the value of preoperative CT was not reported. In Zhou’s article, preoperative hormones values were not mentioned [9].

**Table 3:** Preoperative serum hormones and cytological findings

Author	CT serum levels	CEA serum	Cytological examination	Immunohistochemistry
Sobol	Normal limits	NA	NA	NA
Schmid 1	NA	NA	NA	NA
Schmid 2	NA	NA	NA	NA
Schmid 3	NA	NA	NA	NA
Schmid 4	NA	NA	NA	NA
Redding	8.2 pg/mL	NA	Atypical cells not diagnostic of MTC	Positive for calcitonin
Bockhorn	0.8 pg/mL	Normal limits	Suspicious for MTC or anaplastic cancer	NA
Sand	5.3 pg/mL	NA	NA	NA
Dora	4.0 pg/mL	0.78 ng/mL	Atypias suggesting for malignancy	NA
Wang	38 pg/mL	56.7 ng/mL	Discohesive cells with eccentric nuclei, finely granular chromatin and relatively uniform morphology	Negative for calcitonin
Giovanella	4.7 pg/mL	12.8 ng/mL	Aggregates of elonged cells with finely granular cytoplasm and oval nuclei with coarsely clumped chromatin and nuclear pseudoinclusions	Positive for calcitonin
Alapat	4.0 pg/mL	1.0 ng/mL	Positive for MTC	Positive for MTC
Chernyavsky	2.1 pg/mL	0.5 ng/mL	Findings suspicious for a poorly differentiated carcinoma with neuroendocrine differentiation	Negative for calcitonin
Nakazawa	22 pg/mL	NA	Several solid cell clusters	NA
Frank-Raue 1	2.9 pg/mL	1.3 ng/mL	Suspected malignancy	NA
Frank-Raue 2	≤2 pg/mL	2.1 ng/mL	NA	NA
Frank-Raue 3	< 0.8 pg/mL	2.8 ng/mL	Positive for MTC	NA
Frank-Raue 4	2.6 pg/mL	3.1 ng/mL	NA	NA
Frank-Raue 5	Normal limits	Normal limits	NA	NA
Frank-Raue 6	11 pg/mL	Normal limits	NA	NA
Frank-Raue 7	1.5 pg/mL	1.7 ng/mL	NA	NA
Ismi	5.6 pg/mL	Normal limits	NA	NA
Brutsaert	2.1 pg/mL	3.1 ng/mL	Positive for malignant cells	Positive for calcitonin in isolated cells
Kim	3.7 pg/mL	NA	Positive for MTC	NA
Kasajima	29 pg/mL	NA	Positive for MTC	Negative for calcitonin
Samà 1	7.8 pg/mL	NA	NA	NA
Samà 2	5 pg/mL	NA	NA	NA
Samà 3	< 10 pg/mL	1.8 ng/mL	NA	NA
Samà 4	13 pg/mL	6.3 ng/mL	NA	NA
Parmer	NA	NA	Suspected malignancy	NA
Zhou	NA	NA		

(*CT* calcitonin, *CEA* Carcinoembryonic antigen, *NA* not avaible)

In 12 patients, a value of CEA was detected with a mean value of 7.22 ng/mL and a range of 0.5–56.7 ng/mL. In 4 cases, a value in the normal range was reported and in 13 cases it was not performed preoperatively. (Table 3).

Twenty-three patients underwent fine-needle cytology (FNC) before surgery: six were positive for MTC, seven were suspicious for MTC, a patient was submitted to lymph nodal biopsy that confirmed diagnosis of MTC. FNC of the remaining 25 patients was not reported. Six FNC were studied with IHC: 3 were calcitonin negative, 2 calcitonin positive and one was confirmed to be MTC [13, 17–22].

Among 49 selected patients, 5 patients underwent total thyroidectomy, [4, 5, 14–16] 7 patients total thyroidectomy with central neck compartment lymphadenectomy or/and lateral compartment lymphadenectomy [11, 13, 19, 21–24] and a patient underwent hemithyroidectomy. [10] As regards the remaining cases, the surgery was not specified in the correspondent articles. (Table 4).

**Table 4:** Intraoperative and postoperative findings

Author	Surgery	Tumor grading	Follow-up	Recurrence	CT-IHC	CGRP-IHC	CgA-IHC	Syn-IHC	TG-IHC	CEA-IHC	RET mutation
Sobol	TT	WDMTC	6 month	Lymph nodes, liver and bone	–	–	+	NA	NA	+	NA
Schmid 1	NA	WDMTC	NA	NA	NA	+	+	NA	–	–	NA
Schmid 2	NA	WDMTC	19 month	Lymph nodes	NA	+	+	NA	–	–	NA
Schmid 3	NA	WDMTC	NA	NA	NA	–	+	NA	–	–	NA
Schmid 4	NA	WDMTC	NA	NA	NA	–	+	NA	NA	–	NA
Redding	TT + LYA	WDMTC	43 month	Negative	+	NA	NA	+	NA	+	–
Bockhorn	TT + LYA	PDMTC	NA	NA	NA	NA	+	NA	–	+	+
Sand	TT + LYA	PDMTC	Deceased 6 weeks	NA	NA	NA	NA	NA	NA	NA	NA
Dora	TT	PDMTC	NA	NA	+	NA	+	+	–	NA	–
Wang	NA	WDMTC	12 months	Negative	+	NA	+	NA	–	+	NA
Giovanella	TT + LYA	WDMTC	24 months	NA	+	NA	NA	NA	NA	NA	NA
Alapat	TT + LYA	WDMTC	20 months	Negative	+	NA	+	NA	–	+	NA
Chernyavsky	TT + LYA	WDMTC	12 months	Negative	–	NA	+	+	+	NA	+
Nakazawa	TT	WDMTC	18 months	Negative	NA	NA	+	+	–	NA	NA
Frank-Raue 1	ET	WDMTC	72 months	Negative	+	NA	+	+	–	+	–
Frank-Raue 2	NA	WDMTC	25 months	Pulmonary	+	NA	+	+	–	+	–
Frank-Raue 3	NA	WDMTC	150 months	Lymph modes	+	NA	+	+	–	+	+
Frank-Raue 4	NA	PDMTC	18 months	Local tumor infiltration	+	NA	+	+	–	+	–
Frank-Raue 5	NA	WDMTC	21 months	Lymph node, bone, brain	+	NA	+	+	–	+	+
Frank-Raue 6	NA	PDMTC	21 months	Pulmonary	+	NA	+	+	–	+	+
Frank-Raue 7	NA	PDMTC	36 months	Dead because of pulmonary failure	+	NA	+	+	–	+	+
Ismi	NA	PDMTC	NA	NA	–	NA	+	+	–	NA	NA
Brutsaert	TT + LYA	WDMTC	NA	NA	NA	NA	NA	NA	NA	NA	+
Kim	ET	PDMTC	12 months	Negative	–	NA	+	+	+	–	–
Kasajima	NA	WDMTC	NA	NA	NA	+	+	+	NA	NA	–
Samà 1	TT	NA	120 months	Negative	+	NA	+	NA	NA	+	–
Samà 2	NA	NA	120 months	Negative	NA	NA	NA	NA	NA	NA	NA
Samà 3	NA	NA	36 months	Negative	–	NA	–	NA	NA	–	+
Samà 4	NA	NA	36 months	Negative	+	NA	+	NA	NA	–	–
Parmer	TT	WDMTC	NA	NA	–	NA	+	+	–	+	NA
Zhou	NA	NA	NA	NA	+ 8 cases - 11 cases	NA	+ 18 cases - 1 case	+ 19 cases	+ 5 cases - 14 cases	+ 4 cases - 15 cases	NA in 15 cases - In 4 cases

(TT, total thyroidectomy; ET, emithyroidectomy; LYA, lymphadenectomy; WDMTC, well differentiated MTC; PDMTC, poorly differentiated MTC; NA, not avaible; −, negative; +, positive; IHC, immunohistochemistry; CT, calcitonin; CEA, Carcinoembryonic antigen; CGRP, calcitonin gene related peptide; CgA, chromogranin A; Syn, synaptofisine; TG, thyroglobulin)

### Definitive pathology examination and immunohistochemistry

Definitive pathology detected 18 cases of well differentiated medullary thyroid carcinoma (WDMTC) and 8 cases of poorly differentiated medullary thyroid carcinoma (PDMTC). In the remaining patients the tumor grading was not evaluated. The following markers were tested at immunohistochemistry (IHC): CT, calcitonin gene related peptide (CGRP), CgA, synaptophysin (Syn), thyroglobulin (TG), CEA and RET oncogene mutations. At IHC, CT resulted positive in 21 of the 38 tested cases, while CgA showed a positive stain in all patients examined. RET oncogene mutation was negative in 4 cases of WDMTC, in 3 cases of PDMTC and in 6 cases of unknown differentiation [4, 9, 10, 14, 16, 19, 22–26]. The complete results were showed in Table 4. Mean follow-up was 41 months, with a range from 6 to 150 months. Recurrence was recorded in 7 patients, 2 of whom had multiple organ recurrence [5, 10]. Two patients died due to complications after surgery [10, 11] and seven patients for the disease progression with metastatic localization in lymph nodes, liver, bones, lungs and brain [5, 10, 18].

Microscopical examination and anatomopathological features are reported in Table 5. The neuroendocrine component was detected in 23 cases [4, 5, 9, 12, 22, 23, 25, 26]. The amyloid substance was found in 14 patients, [4, 5, 9, 14, 16, 20] lymph nodes metastasis in 7 cases, [9, 11, 13, 14, 20] thyroid capsular invasion in 15 specimens [9, 11, 13, 14, 18, 20, 24] and vascular tumor thrombus in 7 findings. [9, 11, 14, 18, 20]

**Table 5:** Definitive pathology examination and immunohistochemistry

Authors	Cell morphological characteristics	Neuroendocrine tumor structure	Amyloid substance	Lymph node metastasis	Thyroid capsular invasion	Vascular tumor thrombus
Sobol	Ovoid-to-spindle-shaped in groups divided by fibrous septum	Neurosecretory granules	Focal	NA	NA	NA
Schmid 1	Polygonal and spindle cells	NA	NA	NA	Negative	Negative
Schmid 2	Polygonal and spindle cells	NA	NA	NA	Negative	Negative
Schmid 3	Polygonal and spindle cells	NA	NA	NA	Positive	Positive
Schmid 4	Polygonal and spindle cells	NA	NA	NA	Positive	Positive
Redding	Nets of fairly uniform cells	NA	NA	NA	Negative	NA
Bockhorn	Polyhedral and spindle cells	Positive	NA	NA	Negative	NA
Sand	NA	NA	NA	Positive	Positive	Positive
Dora	Spindle-shaped celss	NA	Positive	Positive	Positive	Positive
Wang	NA	NA	Negative	Positive	Positive	Positive
Giovanella	Elongated cells	NA	NA	NA	NA	NA
Alapat	Spindle-round-polygonal cells	NA	NA	Positive	Positive	Positive
Chernyavsky	Fairly uniform round and polygonal cells	Positive	NA	Negative	Negative	Negative
Nakazawa	“Zellballen” pattern	NA	NA	Negative	Negative	Negative
Frank-Raue 1	NA	NA	NA	NA	NA	NA
Frank-Raue 2	NA	NA	NA	NA	NA	NA
Frank-Raue 3	NA	NA	NA	NA	NA	NA
Frank-Raue 4	NA	NA	NA	NA	NA	NA
Frank-Raue 5	NA	NA	NA	NA	NA	NA
Frank-Raue 6	NA	NA	NA	NA	NA	NA
Frank-Raue 7	NA	NA	NA	NA	NA	NA
Ismi	Atypical cells	Positive	NA	NA	NA	NA
Brutsaert	NA	NA	NA	Negative	Positive	Negative
Kim	NA	Positive	NA	Negative	Negative	Negative
Kasajima	Polygonal-spindle-shaped cells	Positive	NA	Negative	Negative	Negative
Samà 1	Small-spindle cells	Positive	Positive	NA	NA	NA
Samà 2	NA	NA	NA	NA	NA	NA
Samà 3	NA	NA	NA	NA	NA	NA
Samà 4	NA	NA	NA	NA	NA	NA
Parmer	Spindle-round cells	NA	Negative	NA	NA	NA
Zhou	Polygonal cells in 17 cases Spindle cells in 2 cases	Positive in 16 cases Negative in 3 cases	Positive in 11 cases Negative in 8 cases	Positive in 3 cases Negative in 16 cases	Positive in 8 cases Negative in 11 cases	Positive in 1 case Negative in 18 cases

(*NA* not avaible)

## Discussion

MTC is an uncommon and aggressive form of thyroid cancer. Therefore, early identification, surgical resection and careful postoperative surveillance are crucial. The cornerstone in MTC diagnosis and follow-up is the evaluation of CT serum level, which is an index of extreme sensitivity and specificity in case of basal level above of 100 pg/ml. Nevertheless, elevated CT levels may be present in patients affected by autoimmune thyroid disease, in heavy smokers, in end stage renal disease and in patients with pancreas and lung carcinoma [13]. Differential diagnosis is principally formulated on the basis of the pentagastrin stimulating test, which shows an increase in CT above 1000 pg/ml, only in presence of MTC, and throughout the evaluation of CEA and CgA serum levels [1]. New proposed diagnostic tests are the determination of CT on FNC washout fluids, evaluation of serum proCT and calcium stimulation of CT [27–29].

The present review analyze the extremely rare cases of non-secretory MTCs; to date only 49 cases of certified “atypical” MTC have been described in Literature. To the best of our knowledge, this is the first review reporting all cases described in English Literature. Firstly, Sobol et al. reported the case of a 82 years old woman affected by a MTC without CT serum level elevation. The follow up and the relapse identification were achieved through the identification of high CgA serum level, hypothesizing the possibility, in this so-called “chromograninoma”, of an altered co-regulation for genes of CgA and of hormone production [5]. Frank-Raue et al. reported 7 cases of nonsecretory MTC, with a prevalence in his large sporadic MTC population of the 0.83%. Moreover, in Frank-Raue series were reported only a weak or focal immunohistochemical stain in six of the seven cases, even if all cases presented a strong positivity for CgA, suggesting the role of CgA evaluation in addition to CEA in the diagnosis of CT negative MTC [10].

In fact, despite the low or undetectable CT serum level, at IHC in almost the half of the cases reported by the Authors, the tumors presented diffuse or focal positivity for CT and CEA, while was reported a CgA positivity in 41 of the 43 tested patients. As the parafollicular C-cells, NET have a neural crest ectoderm derivation and are present in many organs such as pancreas, lung, small bowel and stomach [30]. The differential diagnosis between MTC and NET is often challenging, because morphologically they have both spindle-shaped or round cells in trabecular arrangements with the presence of amyloid. At IHC, both cells stain positive for CgA, NSE and CEA. Therefore, the evaluation of CT serum level and CT at IHC staining are of paramount importance [22]. On these bases, in the reported atypical MTC cases, it is extremely hard to certainly exclude the diagnosis of a primary or secondary thyroidal NET, leading to a possible therapeutic and prognostic misunderstanding.

The differential diagnosis also includes a distinct type of thyroid neoplasm, the hyalinaising trabecular tumors, which share a similar histological pattern and a positive immunostain for CgA, somatostatin and NSE, conversely present as characteristic and distinctive feature the thyroglobulin hyper-expression [31].

The largest clinical series of nonsecretory MTC was reported by Zhou et al., which identified 19 cases of CT negative MTC among their 158 MTC treated patients with a surprising high prevalence of 12,02% [9]. Zhou, in his study, compared MTC patients vs nonsecretory MTC patients, describing usually larger masses in typical MTC group which were also associated with higher rate of lymph nodes metastasis, thus identifying tumor size as an independent survival indicator. Moreover, the study suggested a better oncological outcome for nonsecretory MTC and that the prognosis was related to the CT serum level. [9] Different findings were reported by Frank-Raue et al., which divided nonsecretory MTC patients into two groups, long-term survival (12,5 years) or rapid progression disease (1,75 years), the latter one characterized by over expression of Ki67 and RET gene mutation [10].

The pathophysiology of CT negative MTC is still not clearly understood. Several reasons have been advocated by eminent Authors to explain this medical dilemma. A possible explanation, reported by several papers, is the possibility of calcitonin assay interferences, or hook effect [14, 32]. The hook effect or prozone effect, is observed when a very high amount of an analyte is present in a sample but the observed value is falsely lowered. The mechanism of this significant negative interference is the capability of a high level of an analyte (antigen) to reduce the concentrations of “sandwich” (antibody 1:antigen:antibody 2) complexes that are responsible for generating the signal by forming mostly single antibody:antigen complexes [33]. Dora et al. have obviated to the hook effect bias by performing 1:10 and 1:100 dilutions of the patient serum [14]. Redding et al. have suggested that tumor cells release different types of serum CT, not all recognized by the same antibodies. Therefore, precursor molecules, aberrant CT produced by abnormal secretory mechanism or a high-dose hook effect in the serum immunoassay can be the possible causes of this phenomenon [19]. Nevertheless, several Authors have demonstrated, through the analysis of the immunohistochemical stain using the same antibodies used for serum calcitonin measurement, that parafollicular cells retain the ability to synthesize but not to secrete CT. In this regard, they hypothesized two possible explanations: the parafollicular cells in MTC undergo to a process of dedifferentiation losing the ability to produce CT or the possibility of a preneoplastic impairment in calcitonin secretion [14]. Alapat et al. too hypothesized an alteration of intracellular secretory pathways in tumor cells [13]. Frank-Raue and Brutsaert hypothesized the possibility in neoplastic C-cells, to secrete an altered proportion of N-proCT, mature CT and C-proCT due to an alternative splicing of the CT gene related peptide (CGRP). Moreover, modern monoclonal antibodies measure only monomeric CT, not detecting premature or aberrant form secreted in atypical MTC [10–24]. Similar findings were reported by Bockhorn et al., which suggested that very aggressive and undifferentiated MTC subtypes lose the ability to produce CT. This theory was supported by the recent American Thyroid Association guidelines for the management of MTC [23]. Sand et al. analyzed the nonsecretory MTC DNA with Southern blot hybridizations and identified a mutation of the calcitonin/CGRP gene which might be responsible of the low or undetectable CT serum level [11]. Nakazawa et al. reached the same conclusions, hypothesizing that the loss of calcitonin production reflects a genetic and/or epigenetic interference with CT/CGRP gene [15].

Schmid et al. proposed a different theory based on the thymic origin for the neuroendocrine thyroid tumors without calcitonin expression. In fact morphologically, MTC cells show histological patterns observed in carcinoid tumor of thymus [18, 34].

A rare thyroidal pathology sharing the same peculiar characteristics such as CT negativity stain at IHC and positivity to CgA and NSE have been identified by Chernyavsky et al., who classified this distinct clinical entity as the CT negative NET of the thyroid. However, this challenging diagnosis should be suspected only in case of positivity of thyroglobulin which is the main distinctive feature of this NET [22].

Indications and surgical treatment does not differ from the typical MTC. Total thyroidectomy and appropriate lymphadenectomy is recommended according to the recent guidelines [35, 36]. Due to the unreliability of serum biomarkers, postoperative surveillance in case of CT negative MTC is unclear. In fact the lack of CT increase in case of disease recurrence makes it necessary to perform, in addition to CT, CEA and CgA evaluations, serial and close imaging tests, including neck US and CT, and MRI of liver and chest, even if the identification of small tumor is hard [14–24]. Frank Raue et al., in addition to the conventional imaging techniques identified the 89% of occult persistent MTC with the selective venous catheterization [10]. Fluorine 18-fluorodeoxyglucose (^18^F-FDG) PET/TC have been proposed in different series as superior than conventional imaging in identifying relapse or disease persistence [35]. Conversely, other Authors considered ^18^F-FDG as an expensive technique not available in all centers, with a variable sensitivity ranging from 50 to 85% and not able to detect small masses [14].

Therefore, in order to better follow up patients after surgical treatment, new and alternative biomarkers are claimed in nonsecretory MTC. Between them the most reliable and promising indicators are ProCT and CGRP. ProCT is the precursor of CT, with a diagnostic accuracy comparable to CT in terms of identification of primary tumor, extrathyroidal extension and meatastases [37]. It is a very stable protein, easy to manage in pre-analytical level and with an in vivo half-life of 24 h [38]. With these positive features, Pro-CT has a great potential to replace serum CT as a new standard of care in the management of nonsecretory MTC. CGRP is a neuropeptide normally secreted by neurons and expressed both in MTC and in non neoplastic C-Cells. It is generated from the alternative RNA splicing of the CLC-A gene, which encodes for CT and CGRP. Its overexpression is not univocally linked with tumoral growth but is consistent with C-Cell origin, especially in case of co-expression TTF-1 and PAX-8. According to Brutsaert et al., among 18 patients diagnosed of MTC, CGRP was expressed in 66% in primary localization and in 73% of the metastases [24]. Moreover, CGRP is not expressed in follicular lineage and might be used to differentiate thyroidal NET from nonsecretory MTC [16].

## Conclusions

CT negative MTC is an extremely rare pathology. The pathophysiology of CT negative MTC is still not clearly understood. Several reasons have been advocated by eminent Authors to explain this medical dilemma, the altered cellular secretion mechanisms, the production of aberrant CT precursors not recognized by the testing antibodies, the hook effect, an ectopic thymic origin, unfortunately without reaching any definitive conclusions. Due to the lack of elevation of serum markers as CT and CEA, atypical MTC is often diagnosed at an advanced stage, leading moreover to a challenging surveillance. The prognosis reported is extremely variable, differing from long term survival and rapid tumor progression in case of poorly differentiated diagnosis, high Ki67 expression and RET mutation. Further studies are needed, to better define options for management of non secretory MTCs and to identify new and reliable biomarkers associated to diagnosis and relapse of this medical dilemma.

## Abbreviations

CEA: Carcinoembryonic antigen
CgA: Chromogranin A
CGRP: Calcitonin gene related peptide
CT: Calcitonin
CT: Computed tomography
DFS: Disease-free survival
ET: Emithyroidectomy
FNC: Fine needle aspiration cytology
IHC: Immunohistochemistry
LYA: Lymphadenectomy
MTC: Medullary thyroid carcinoma
NET: Neuroendocrine tumor
NSE: Neuron specific enolase
OS: Overall survival
pro-CT: Pro-calcitonin
Syn: Synaptophysin
TG: Thyroglobulin
TT: Total thyroidectomy
US: Ultrasonography

## Authors’ information

Gambardella Claudio and Mauriello Claudio are PhD student in Medical, Clinical and Sperimental Sciences at Univerity of Campania “Luigi Vanvitelli” – Naples, Italy.
